# Outcomes of acute ischemic stroke in kidney transplant recipients: An analysis of US Nationwide inpatient sample

**DOI:** 10.1515/tnsci-2022-0247

**Published:** 2022-09-24

**Authors:** Lei Zhang, Zhipeng Wang, Jingcheng Lv, Mengmeng Zheng, Yichen Zhu

**Affiliations:** Department of Urology, Beijing Friendship Hospital, Capital Medical University, No. 95 Yong’an Road, Beijing, 100050, China

**Keywords:** acute ischemic stroke, kidney transplant, chronic kidney disease, dialysis, nationwide inpatient sample

## Abstract

A kidney transplant is often the treatment of choice for end-stage kidney disease, compared with a lifetime on dialysis. Kidney transplant recipients (KTRs) have a reduced risk for new strokes than patients with chronic kidney disease (CKD) G5 treated by dialysis (CKD G5D). However, the benefit of Kidney transplant on post-stroke hospitalization outcomes has not been well studied. This study aimed to evaluate the outcomes of hospitalization after acute ischemic stroke (AIS) in KTRs and patients with CKD G5D. This retrospective study used patient data from the US Nationwide Inpatient Sample database. From 2005 to 2018, patients hospitalized with AIS were classified into 3 groups, including KTRs (*n* = 1,833), patients with CKD G5D (*n* = 26,767), and those without CKD (CKD-free, *n* = 986,945). Patients with CKD G1–G4 or unspecified stage, and graft failure requiring dialysis were excluded. In-hospital mortality, medical complications, transfer to nursing homes, and length of stay (LOS) were compared. Compared to CKD-free group, KTRs had no significant higher risks for in-hospital mortality, transfer to nursing homes, and LOS, but a greater risk for medical complications after adjusting for relevant factors. CKD G5D group had higher risks for in-hospital mortality (adjusted odds ratio (aOR): 2.04, 95% confidence interval (CI): 1.93–2.15), medical complications (aOR: 1.49, 95% CI: 1.45–1.54), and transfer to nursing homes (aOR: 1.10, 95% CI: 1.07–1.13), and a 0.07 day (95% CI: 0.06–0.08) longer LOS than CKD-free group. In conclusion, the outcomes of AIS hospitalization were more favorable in KTRs as compared with CKD G5D. Furthermore, the risks for in-hospital mortality, transfer to long-term care facilities, and LOS were not significantly different between KTRs and CKD-free patients.

## 1 Introduction

Chronic kidney disease (CKD) is an independent risk factor for stroke [1,2,3]. Suggested mechanisms include dysregulated cerebral blood flow, endothelial dysfunction, accelerated atherosclerosis, chronic inflammation, and so on [1,2]. The incidences of stroke are more than 2-fold and 3.5-fold higher in patients with CKD and end-stage kidney disease (ESKD) than in general population [4], respectively. Risk also varies by CKD treatment, with a risk peak for initiation of dialysis, but dropping after the first month of treatment [5,6]. Patients with ESKD treated by dialysis have an 8-fold higher incidence of acute ischemic stroke (AIS) compared to non-dialysis patients [7]. Furthermore, some studies have demonstrated that patients with CKD have poorer outcomes and higher mortality after stroke [1,2,4,6,7]. The standardized mortality ratio in patients with CKD was reported around 3-fold higher than the general population [4,8]. The risk of stroke death was higher in patients who initiated dialysis at an younger age [9].

Kidney transplant (KT) is often recognized as the treatment of choice for ESKD. Previous studies showed kidney transplant recipients (KTRs) had an all-cause mortality benefit [10] and a significantly lower risk of stroke [11,12,13,14] as compared with those with CKD Stage G5 treated by long-term dialysis; and the risk even reduced to similar level as CKD-free population [14,15]. However, in KTRs, whether the benefit extends to clinical outcomes when strokes occur has not been well examined.

The retrospective study aimed to evaluate the clinical outcomes of AIS hospitalization when comparing between KTRs, patients of CKD G5D, and those without CKD, using a nationally representative database.

## 2 Methods

### 2.1 Data source

This population-based, retrospective observational study extracted all data from the US Nationwide Inpatient Sample (NIS) database, which is the largest all-payer, continuous inpatient care database in the United States, including about eight million hospital stays each year [16]. The database is administered by the Healthcare Cost and Utilization Project (HCUP) of the US National Institutes of Health (NIH). Patient data include primary and secondary diagnoses, primary and secondary procedures, admission and discharge status, patient demographics, expected payment source, duration of hospital stay, and hospital characteristics (i.e., bed size/location/teaching status/hospital region). All admitted patients are initially considered for inclusion. The continuous, annually updated NIS database derives patient data from about 1,050 hospitals from 44 States in the US, representing a 20% stratified sample of US community hospitals as defined by the American Hospital Association.


**Ethics statement:** All data were obtained through request to the Online HCUP Central Distributor (available at: https://www.distributor.hcup-us.ahrq.gov/), which administers the database (certificate # HCUP-1K60EVP88). This study conforms to the NIS data-use agreement with HCUP. Because this study analyzed secondary data from the NIS database, patients and the public were not involved directly. Since all data in the NIS database are de-identified, the requirement for informed consent was also waived.

### 2.2 Study population

Data of hospitalized adults aged 18–85 years or older who had a principal diagnosis of ischemic stroke between 2005 and 2018 were identified in the NIS database through the International Classification of Diseases, Ninth and Tenth Revision, Clinical Modification (ICD-9-CM and ICD-10-CM) codes: 433, 434, 436, and I63. Exclusion criteria were: kidney graft failure requiring dialysis (ICD-9-CM: V42.0 or 996.81 combined with ICD-9-PCS 39.95 or 54.98; ICD-10-CM: Z94.0 or T8610 combined with 5A1D70Z, 5A1D80Z, 5A1D90Z, or 3E1M39Z); CKD G1–G4 and unspecified stage CKD (585.1–585.4, 585.9, N18.1–18.4, or N18.9); patients admitted to the hospital electively (non-emergently), and no information of study outcome variables (i.e., mortality, LOS, and transfer to nursing home/long-term facilities). The patient cohort was further classified into three groups for further comparisons: CKD G5D, i.e., patients with codes of CKD G5 treated by dialysis (ICD-9-CM: 585.5, 585.6 or ICD-9-PCS: 39.95, or 54.98 excluding the codes of acute kidney injury, and ICD-10-CM: N18.5, N18.6, or ICD-10-PCS: 5A1D70Z, 5A1D80Z, 5A1D90Z, or 3E1M39Z excluding the codes of acute kidney injury); KTRs (V42.0 and Z94.0); and CKD-free, i.e., none of the abovementioned codes.

### 2.3 Study outcomes

Study outcomes were: (1) in-hospital mortality; (2) transfer to nursing homes or long-term care facilities; (3) the occurrence of major medical complications; and (4) LOS.

Major medical complications with applicable ICD-9 and ICD-10 codes included: intracranial hemorrhage, acute myocardial infarction (AMI), respiratory complication, pneumonia, sepsis, infection, deep vein thrombosis (DVT)/pulmonary embolism (PE), received mechanical ventilation and parenteral nutrition.

### 2.4 Covariates

Patients’ characteristics included age, gender, race, household income level, and insurance status (primary payer). Reperfusion treatment including thrombolysis, endovascular thrombectomy, or none, comorbidities and Charlson’s Comorbidity Index (CCI) were identified using ICD-9 and ICD-10 diagnostic codes. Hospital-related characteristics (year of hospitalization, bed size, location/teaching status, hospital region, and annual caseload) were also extracted from the database as part of the comprehensive data available for all participants.

### 2.5 Statistical analysis

Comparisons of the continuous data were performed using the Analysis of Variance (ANOVA) and presented as mean value ± standard deviation (SD). Comparisons of the categorical data were performed using the chi-square test and presented as *n* (%). Univariable and multivariable logistic regressions and linear regressions were utilized to determine the associations between study variables and in-hospital mortality, transfer to nursing homes and long-term care facilities, medical complications, and length of stay. A two-sided *P*-value of <0.05 was regarded as statistically significant. Data management and statistical analyses were conducted by using SAS version 9.4 software (SAS Institute, Inc.).

## 3 Results

**Figure 1:**
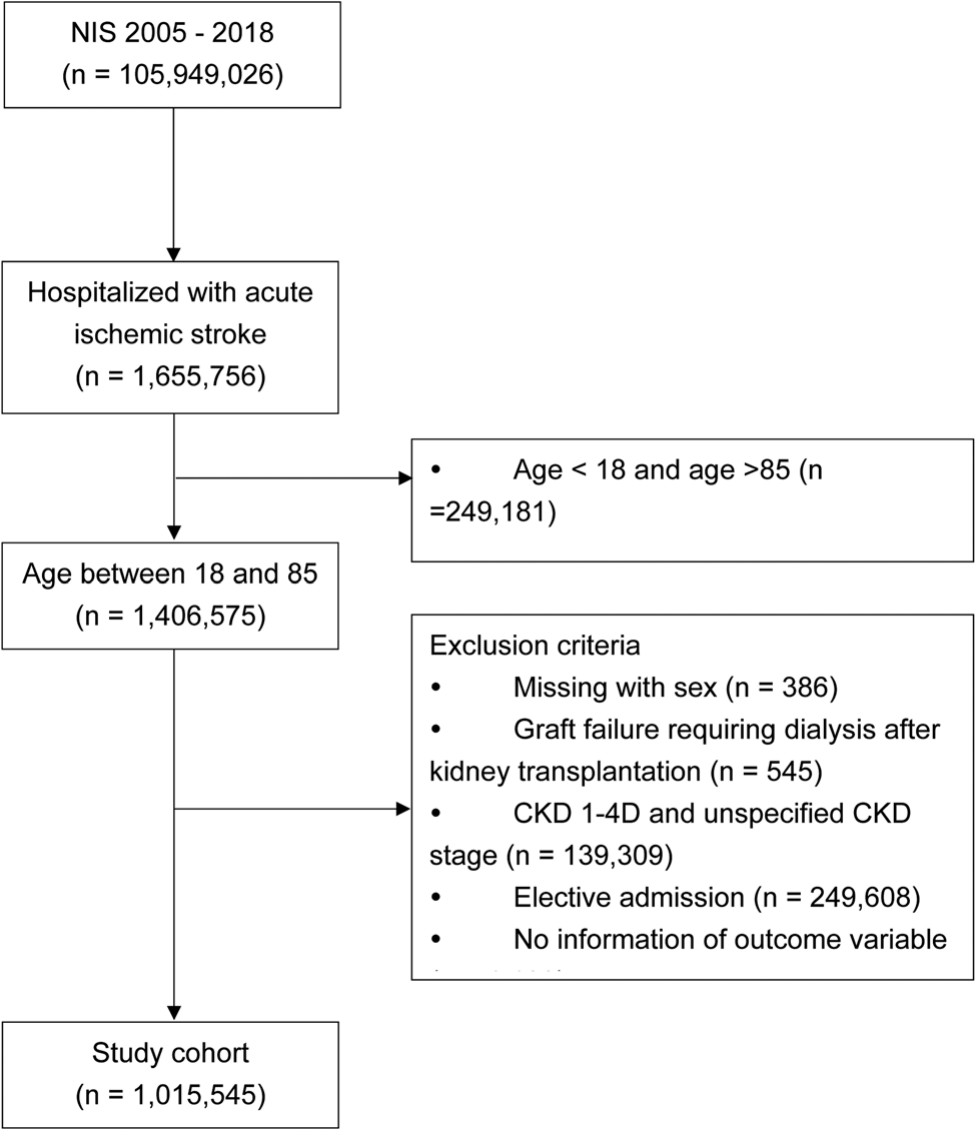
Flow diagram of study selection.

During 2005–2018 in the NIS database, totally 1,655,756 hospitalized patients 18–85 years of age had a principal diagnosis of AIS. After exclusion for kidney graft failure requiring dialysis, patients with CKD G1–G4 or unspecified CKD stage, admitted to hospital electively, and no information of outcome and main study variables, there remained 1,015,545 patients included in the subsequent analyses. The flow diagram of study selection is shown in Figure 1.

### 3.1 Baseline characteristics of patients hospitalized for AIS

Table 1 summarizes the characteristics of all patients hospitalized for AIS, grouped by CKD and KT status: CKD-free: *n* = 986,945, CKD G5D: *n* = 26,767, and KTR: *n* = 1,833. Of the study cohort, the mean age was 66.56 and 50.74% were males. Patients in the KTR group were the youngest (CKD-free: 66.61 ± 12.75 y/o, CKD G5D: 64.95 ± 12.72 y/o, KTR: 61.70 ± 10.95 y/o, respectively; *p*-value <0.001). Patients in the CKD G5D group had the greatest proportion of female (CKD-free: 49.17%; CKD G5D: 53.17%; KTR: 42.23%; *p*-value < 0.001), black race (CKD-free: 16.86%; CKD G5D: 36.30%; KTR: 21.76%; *p*-value < 0.001), lowest household income (CKD-free: 30.55%; CKD G5D: 38.25%; KTR: 26.69%; *p*-value < 0.001), insurance covered by Medicare/Medicaid (CKD-free: 67.65%; CKD G5D: 86.15%; KTR: 76.19%; *p*-value <0.001), and no reperfusion treatment (CKD-free: 91.55%; CKD G5D: 94.86%; KTR: 93.67%; *p*-value <0.001).

**Table 1:** Baseline characteristics of patients hospitalized for AIS

	Total (*n* = 1,015,545)	CKD-free (*n* = 986,945)	CKD G5D (*n* = 26,767)	KTR (*n* = 1,833)	*P*-value
Age	66.56 ± 12.75	66.61 ± 12.75	64.95 ± 12.72	61.70 ± 10.95	**<0.001**
<50	104,726 (10.60)	101,307 (10.55)	3,165 (12.00)	254 (13.87)	**<0.001**
50–64	305,523 (30.91)	296,044 (30.83)	8,690 (32.95)	789 (43.09)	
65–74	275,389 (27.86)	266,815 (27.79)	7,996 (30.32)	578 (31.57)	
75–84	302,786 (30.63)	296,055 (30.83)	6,521 (24.73)	210 (11.47)	
Gender					**<0.001**
Female	500,245 (49.26)	485,239 (49.17)	14232 (53.17)	774 (42.23)	
Male	515,300 (50.74)	501,706 (50.83)	12,535 (46.83)	1,059 (57.77)	
Race					**<0.001**
White	613,341 (68.39)	602,252 (69.13)	10,098 (42.16)	991 (60.24)	
Black	155,904 (17.39)	146,852 (16.86)	8,694 (36.30)	358 (21.76)	
Hispanic	74,156 (8.27)	70,529 (8.10)	3,442 (14.37)	185 (11.25)	
Others	53,371 (5.95)	51,542 (5.92)	1,718 (7.17)	111 (6.75)	
Household income					**<0.001**
Q1	305,574 (30.75)	295,062 (30.55)	10,034 (38.25)	478 (26.69)	
Q2	262,141 (26.38)	255,006 (26.41)	6,688 (25.50)	447 (24.96)	
Q3	233,503 (23.50)	227,502 (23.56)	5,516 (21.03)	485 (27.08)	
Q4	192,529 (19.37)	188,155 (19.48)	3,993 (15.22)	381 (21.27)	
Primary payer					**<0.001**
Medicare/Medicaid	691,879 (68.25)	667,464 (67.75)	23,020 (86.15)	1,395 (76.19)	
Private including HMO	236,025 (23.28)	232,682 (23.62)	2,965 (11.10)	378 (20.64)	
Self-pay/no charge/others	85,871 (8.47)	85,078 (8.64)	735 (2.75)	58 (3.17)	
Year of hospitalization					**<0.001**
2015–2018	360,714 (35.52)	350,589 (35.52)	9,549 (35.67)	576 (31.42)	
2010–2014	364,382 (35.88)	353,840 (35.85)	9,905 (37.00)	637 (34.75)	
2005–2009	290,449 (28.60)	282,516 (28.63)	7,313 (27.32)	620 (33.82)	
Comorbidities					
Coronary artery disease	258,862 (25.49)	247,983 (25.13)	10,302 (38.49)	577 (31.48)	**<0.001**
Congestive heart failure	114,537 (11.28)	105,890 (10.73)	8,421 (31.46)	226 (12.33)	**<0.001**
Diabetes	362,217 (35.67)	343,605 (34.82)	1,7451 (65.20)	1,161 (63.34)	**<0.001**
Hypertension	777,493 (76.56)	755,973 (76.60)	20,145 (75.26)	1,375 (75.01)	**<0.001**
Hyperlipidemia	536,619 (52.84)	524,482 (53.14)	11,208 (41.87)	929 (50.68)	**<0.001**
COPD	148,232 (14.60)	143,933 (14.58)	4,174 (15.59)	125 (6.82)	**<0.001**
Atrial fibrillation	178,771 (17.60)	173,312 (17.56)	5,123 (19.14)	336 (18.33)	**<0.001**
Obesity	105,607 (10.40)	102,748 (10.41)	2,727 (10.19)	132 (7.20)	**<0.001**
Drug abuse	84,652 (8.34)	83,372 (8.45)	1,221 (4.56)	59 (3.22)	**<0.001**
CCI					**<0.001**
0	260,534 (25.65)	257,599 (26.10)	2,656 (9.92)	279 (15.22)	
1–3	632,879 (62.32)	615,159 (62.33)	16,518 (61.71)	1,202 (65.58)	
4–6	103,413 (10.18)	96,284 (9.76)	6,816 (25.46)	313 (17.08)	
7+	18,719 (1.84)	17,903 (1.81)	777 (2.90)	39 (2.13)	
**Hospital characteristics**					
Hospital bed size					**<0.001**
Large (>450)	625,143 (61.78)	606,747 (61.70)	17,191 (64.48)	1,205 (65.81)	
Medium (250–450)	260,430 (25.74)	253,255 (25.75)	6,741 (25.28)	434 (23.70)	
Small (<250)	126,254 (12.48)	123,333 (12.54)	2,729 (10.24)	192 (10.49)	
Location/teaching status					**<0.001**
Urban teaching	549,023 (54.26)	532,480 (54.15)	15,322 (57.47)	1,221 (66.68)	
Urban nonteaching	360,709 (35.65)	350,572 (35.65)	9,636 (36.14)	501 (27.36)	
Rural	102,095 (10.09)	100,283 (10.20)	1,703 (6.39)	109 (5.95)	
Hospital region					**<0.001**
Northeast	180,427 (17.77)	175,699 (17.80)	4,384 (16.38)	344 (18.77)	
Midwest	220,620 (21.72)	214,767 (21.76)	5,436 (20.31)	417 (22.75)	
South	430,982 (42.44)	418,455 (42.40)	11,829 (44.19)	698 (38.08)	
West	183,516 (18.07)	178,024 (18.04)	5,118 (19.12)	374 (20.40)	
**Hospital annual caseload of AIS (cases)**					**<0.001**
Q1 (1–38)	257,439 (25.35)	251,118 (25.44)	5,923 (22.13)	398 (21.71)	
Q2 (39–84)	249,652 (24.58)	242,557 (24.58)	6,621 (24.74)	474 (25.86)	
Q3 (85–190)	254,357 (25.05)	246,703 (25.00)	7,210 (26.94)	444 (24.22)	
Q4 (191–1,336)	254,097 (25.02)	246,567 (24.98)	7,013 (26.20)	517 (28.21)	

AIS, acute ischemic stroke; CCI, Charlson’s Comorbidity Index; COPD, chronic obstructive pulmonary disease; CKD G5D, chronic kidney disease G5 treated by dialysis; KTRs, kidney transplant recipients.

Significant values (*p* < 0.05) are shown in bold.

Clinical outcomes of AIS hospitalizations are shown in Table 2. Among the patient groups, the CKD G5D group had the greatest percentage of in-hospital mortality (CKD-free: 3.37%; CKD G5D: 7.36%; KTR: 3.98%; *p*-value < 0.001), transfer to nursing homes and long-term care facilities (CKD-free: 35.45%; CKD G5D: 41.36%; KTR: 32.79%; *p*-value < 0.001), and occurrence of any medical complication (CKD-free: 17.55%; CKD G5D: 28.98%; KTR: 19.01%; *p*-value < 0.001). In addition, the CKD G5D group also had the longest LOS (CKD-free: 4.80 ± 5.98 days ; CKD G5D: 7.57 ± 9.5 days5; KTR: 5.08 ± 10.32 days; *p*-value < 0.001).

**Table 2:** Clinical outcomes of patients hospitalized for AIS

	Total (*n* = 1,015,545)	CKD-free (*n* = 986,945)	CKD G5D (26,767)	KTRs (*n* = 1,833)	*P*-value
In-hospital mortality					**<0.001**
No	980,263 (96.53)	953,705 (96.63)	24,798 (92.64)	1,760 (96.02)	
Yes	35,282 (3.47)	33,240 (3.37)	1,969 (7.36)	73 (3.98)	
Transfer to nursing homes/long-term care facilities					**<0.001**
No	618,713 (60.92)	603,826 (61.18)	13,728 (51.29)	1,159 (63.23)	
Yes	361,550 (35.60)	349,879 (35.45)	11,070 (41.36)	601 (32.79)	
Medical complication	181,328 (17.86)	173,205 (17.55)	7,758 (28.98)	365 (19.91)	**<0.001**
Intracranial hemorrhage	28,092 (2.77)	27,318 (2.77)	713 (2.66)	61 (3.33)	0.201
AMI	19,156 (1.89)	18,033 (1.83)	1,083 (4.05)	40 (2.18)	**<0.001**
Respiratory complication	32,342 (3.18)	30,380 (3.08)	1,894 (7.08)	68 (3.71)	**<0.001**
Pneumonia	28,447 (2.80)	26,825 (2.72)	1,576 (5.89)	46 (2.51)	**<0.001**
Sepsis	42,034 (4.14)	39,198 (3.97)	2,749 (10.27)	87 (4.75)	**<0.001**
Infection	78,151 (7.70)	75,610 (7.66)	2,396 (8.95)	145 (7.91)	**<0.001**
DVT/PE	18,023 (1.77)	17,024 (1.72)	950 (3.55)	49 (2.67)	**<0.001**
Mechanical ventilation	24,173 (2.38)	22,766 (2.31)	1,359 (5.08)	48 (2.62)	**<0.001**
Parenteral nutrition	309 (0.03)	295 (0.03)	14 (0.05)	0 (0.00)	0.088
LOS ^a^	4.87 ± 6.12	4.80 ± 5.98	7.57 ± 9.55	5.08 ± 10.32	**<0.001**

AMI, acute myocardial infarction; CKD, chronic kidney disease; CKD G5D, chronic kidney disease G5 treated by dialysis; DVT/PE, deep vein thrombosis/pulmonary embolization; KTRs, kidney transplant recipients; LOS, length of stay.

Significant values (*p* < 0.05) are shown in bold.

### 3.2 Associations between clinical outcomes and KTR, CKD G5D vs CKD-free

The results of logistic regression and linear regression are presented in Table 3. After adjusting for relevant confounders, CKD G5D was significantly and independently associated with an increased risk for in-hospital mortality (adjusted odds ratio (aOR) = 2.04, 95% CI = 1.93–2.14), any medical complication (aOR = 1.49, 95% CI = 1.45–1.54), and transfer to nursing homes or long-term care facilities (aOR = 1.10, 95% CI = 1.07–1.13) as compared with CKD-free. CKD G5D was also associated with a longer LOS (*β* = 0.07, 95% CI = 0.06–0.08) as compared with CKD-free. On the other hand, after adjustment, KTR was significantly associated with an increased risk of any medical complication (aOR = 1.20, 95% CI = 1.05–1.36) as compared with CKD-free.

**Table 3:** Association between KTR, CKD G5D vs CKD-free and clinical outcomes in patients hospitalized for AIS

	In-hospital mortality		Any medical complications		Transfer to nursing homes/long-term care facilities		LOS (days)	
	OR (95% CI)	aOR (95% CI)	OR (95% CI)	aOR (95% CI)	OR (95% CI)	aOR (95% CI)	*β* (95% CI)	adjusted *β* (95% CI)
CKD-free	1.00	1.00	1.00	1.00	1.00	1.00	Ref.	Ref.
CKD G5D	**2.37 (2.25, 2.50)**	**2.04 (1.93, 2.15)**	**1.95 (1.89, 2.00)**	**1.49 (1.45, 1.54)**	**1.43 (1.39, 1.47)**	**1.10 (1.07, 1.13)**	**0.15 (0.14, 0.15)**	**0.07 (0.06, 0.08)**
KTRs	1.18 (0.91, 1.53)	1.23 (0.95, 1.60)	**1.21 (1.07, 1.37)**	**1.20 (1.05, 1.36)**	0.93 (0.84, 1.04)	0.89 (0.80, 1.00)	−0.01 (−0.03, 0.02)	−0.01 (−0.04, 0.01)

CKD, chronic kidney disease; CKD G5D, chronic kidney disease G5 treated by dialysis; KTRs, kidney transplant recipients; OR, odds ratio; aOR, adjusted OR.

Multivariable models are adjusted for age, gender, race, household income, primary payer, reperfusion, comorbidities, year of hospitalization, hospital bed size, location/teaching status, hospital region, and hospital annual caseload of ischemic stroke.

Significant values (*p* < 0.05) are shown in bold.

### 3.3 Associations between medical complications and KTR, CKD G5D vs CKD-free

**Figure 2:**
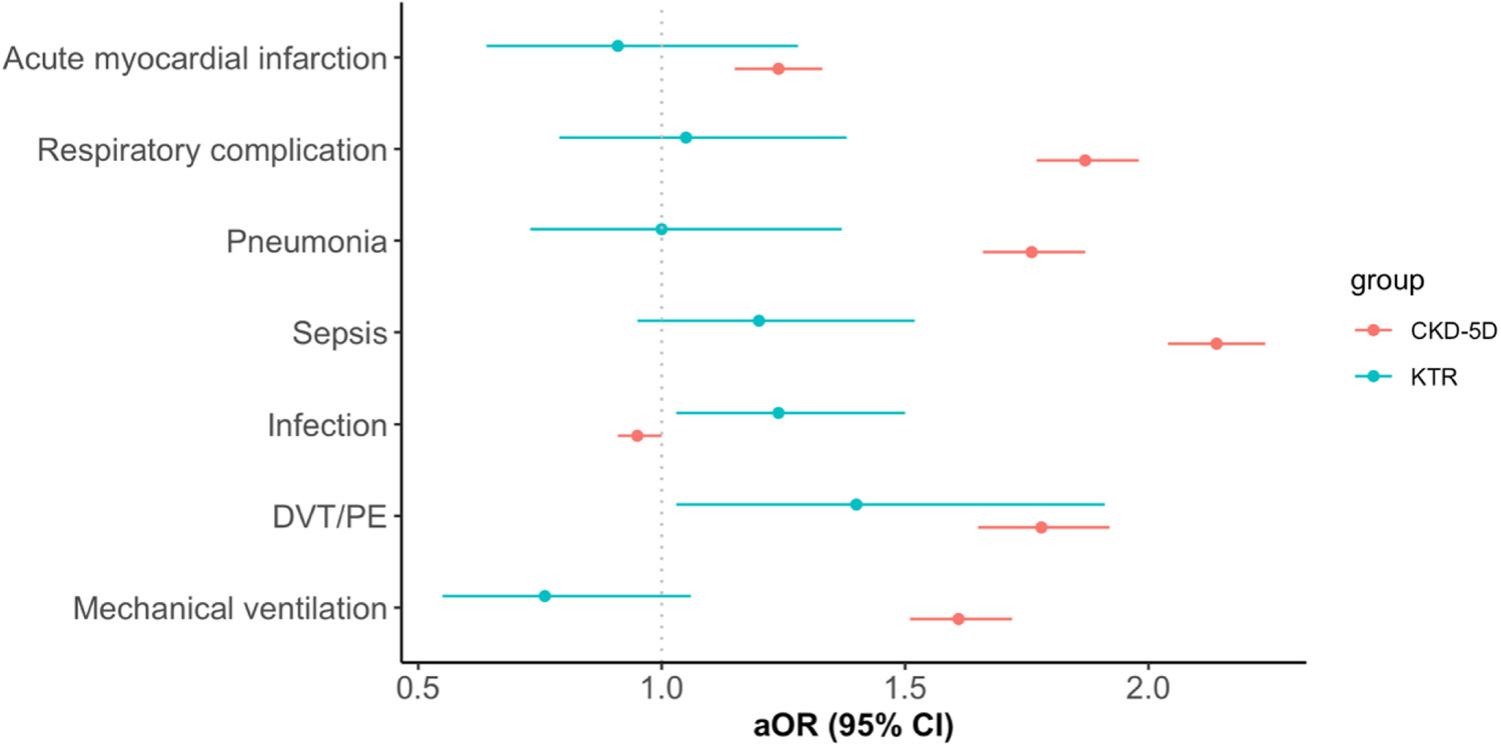
Impact of KTR and CKD G5D on medical complications of stroke hospitalizations. All models are adjusted by age, gender, race, household income, primary payer, reperfusion, comorbidities, year of hospitalization, hospital bed size, location/teaching status, hospital region, and hospital annual caseload of ischemic stroke.

Figure 2 demonstrates the associations between each medical complication, KTR and CKD G5D (versus CKD-free) during AIS admission. CKD G5D was associated with increased risk for AMI, respiratory complication, pneumonia, sepsis, DVT/PE, and mechanical ventilation than CKD-free. There were no significant differences in the risk for AMI, respiratory complication, pneumonia, sepsis, and mechanical ventilation between KTR and CKD-free. However, KTR had a greater odds ratio for infection than that of CKD G5D.

## 4 Discussion

In patients with AIS hospitalizations, as compared with patients without CKD, CKD G5D was associated with increased risks of worse clinical outcomes in terms of in-hospital mortality, medical complications, transfer to nursing homes/long-term care facilities, and longer LOS. However, compared with patients without CKD, there is no significant risk increase among KTRs in in-hospital mortality, transfer to nursing homes, and long-term care facilities, or longer LOS. Among the medical complications, while CKD G5D posed a greater risk for AMI, respiratory complication, pneumonia, sepsis, and mechanical ventilation, we observed no significant risk differences on such outcomes between KTRs and CKD-free patients. The findings generally indicate that outcomes of AIS hospitalizations are more positive in KTRs than in patients of CKD G5D.

Our study showed that the risk of in-hospital mortality was significantly greater in CKD G5D group when compared with CKD-free group, but was not significantly higher in KTRs than in CKD-free group. This is similar with literature that reported KTRs displayed a significantly reduced risk for mortality for AMI as compared with patients of CKD G5D [17,18]. Given that AIS shares similar pathophysiology with AMI through atherosclerosis, the mechanisms proposed in the prior studies could also be applied in the present one, including the status of atherosclerosis, vascular calcification, artery stiffness, and general inflammation [1,2,3,17]. For example, ultrasonography studies have showed significantly larger plaque size, carotid intima-media thickness, and carotid artery stenosis in CKD patients compared to CKD-free group [19,20]. A PET-CT scan study further revealed that cerebral blood flow fell by 10 ± 15% in all brain regions during dialysis [21]. These altogether accumulate an increasing risk of ischemic stroke in ESKD patients requiring dialysis, while the risk from dialysis may be corrected at least partially in KTRs. However, risk of all-cause mortality was still higher in KTRs with a stroke history than those without [22].

We observed no significant risk increase in CKD G5D patients than in CKD-free patients, whereas the infection risk is significantly higher in KTRs than CKD-free. This is probably due to the immunosuppression state in KTRs because they must take immunosuppressant drugs for life-long. The sources of infection differ in different stages after transplantation [23]. Infection-related mortality is significantly higher in KTRs than general population [24], with a downtrend over the past 20 years [24,25]. Persistent monitoring and accurate diagnosis after infection as soon as possible are the recommended strategies for good prognosis of KTRs.

The present analyses found KT has benefits in stroke-related outcomes than that in CKD G5D patients. Studies also asserted a superior outcome and health-related quality of life in KTR patients than in CKD G5D patients in general [26,27,28]. This may attribute to improved physical function in KTRs than in CKD G5D patients [27]. A lower risk for transfer to long-term care facilities as observed in the present analysis also indicated better physical function and agrees with the previous findings that documented a more independent life after KTRs as compared with CKD G5D.

## 5 Strengths and limitations

The strength of the present study is the use of a very large sample that represents a nationwide population, adding credence to the results. Important limitations include the possibility of coding errors during the use of the ICD-9 and ICD-10 coding systems for defining KTR and CKD. For instance, although the ICD-9 diagnosis for CKD is highly specific, the reported sensitivity is only around 80% [29], therefore some patients with milder degrees of CKD may have been misclassified as not having CKD. Because this coding system was also used for comorbidities and complications, the severity of such comorbidities and complications is unknown. Possible confounding variables not collected by the NIS could not be included in the analyses. The study lacks follow-up data after discharge, precluding the evaluation of late morbidity and mortality. Besides, the number of inpatients transferred to acute inpatient rehabilitation were not included in the study. We might underestimate the risk as patients completed acute inpatient rehabilitation could have contributed to improvement in function among many of the patient population. In addition, the relation between AIS and CKD stage 1–4 after KTR could not be further explored since the present study lacks laboratory data.

## 6 Conclusion

In general, outcomes of hospitalization for AIS are more favorable in KTRs as compared with patients of CKD G5D. The risk for in-hospital mortality and transfer to long-term care showed no significant differences between KTRs and patients without CKD admitted for AIS.
